# Supplementary material for: Human Rad51 Protein Requires Higher Concentrations of Calcium Ions for D-Loop Formation than for Oligonucleotide Strand Exchange
Source: Int J Mol Sci. 2024 Mar 24;25(7):3633. doi: 10.3390/ijms25073633 (PMC11011376; doi:10.3390/ijms25073633)
Supplement: Supplementary file 1 [file ijms-25-03633-s001.zip › ijms-2869372-supplementary.pdf]

Supplementary figures

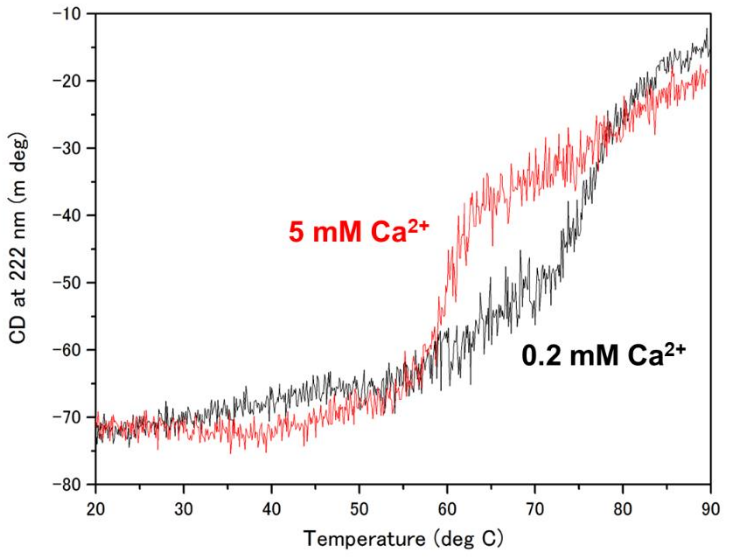

Figure S1. Ca^{2+} concentration dependence of thermal denaturation of the ATP/HsRad51/poly(dT) presynaptic complex. Thermal denaturation was performed as described in the text with 0.2 mM and 5 mM Ca^{2+}.
